# Quantifying Radiation Belt Electron Loss Processes at *L* < 4

**DOI:** 10.1029/2022JA030756

**Published:** 2022-10-22

**Authors:** S. G. Claudepierre, Q. Ma, J. Bortnik

**Affiliations:** ^1^ Department of Atmospheric and Oceanic Sciences UCLA Los Angeles CA USA; ^2^ Center for Space Physics Boston University Boston MA USA

**Keywords:** radiation belt lifetime, coulomb energy drag, lightning generated whistler, pitch angle diffusion, electron decay and loss, wave‐particle interaction

## Abstract

We present a comprehensive analysis of the processes that lead to quasilinear pitch‐angle‐scattering loss of electrons from the *L* < 4 region of the Earth's inner magnetosphere during geomagnetically quiet times. We consider scattering via Coulomb collisions, hiss waves, lightning‐generated whistler (LGW) waves, waves from ground‐based very‐low‐frequency (VLF) transmitters, and electromagnetic ion cyclotron (EMIC) waves. The amplitude, frequency, and wave normal angle spectra of these waves are parameterized with empirical wave models, which are then used to compute pitch‐angle diffusion coefficients. From these coefficients, we estimate the decay timescales, or lifetimes, of 30 keV to 4 MeV electrons and compare the results with timescales obtained from in‐situ observations. We demonstrate good quantitative agreement between the two over most of the *L* and energy range under investigation. Our analysis suggests that the electron decay timescales are very sensitive to the choice of plasmaspheric density model. At *L* < 2, where our theoretical lifetimes do not agree well with the observations, we show that including Coulomb energy drag (ionization energy loss) in our calculations significantly improves the quantitative agreement with the observed decay timescales. We also use an accurate model of the geomagnetic field to provide an estimate of the effect that the drift‐loss cone has on the theoretically calculated electron lifetimes, which are usually obtained using an axisymmetric dipole field.

## Key Points

- Coulomb energy drag is an important electron loss process at *L* ≤ 2 and should not be neglected in theoretical and numerical treatments
- Electron decay timescales in the *L* < 4 region are very sensitive to the choice of plasmaspheric density model
- Explicitly incorporating lightning‐generated whistler waves into our lifetime calculations improves the quantitative agreement with the observations at *L* ≈ [1.8, 3.2]

## 1 Introduction

The high‐energy tail of the plasma in near‐Earth space is trapped by the geomagnetic field, forming the Van Allen radiation belts that encircle the Earth. Various physical processes can rapidly accelerate these charged particles to prodigious energies, in excess of one megaelectron volt (MeV), on timescales of 1 day or less (Li & Hudson, 2019, and references therein). Particles are removed from the belts on similar timescales via drift to the magnetopause and through interactions with the rich variety of plasma waves that populate the inner magnetosphere and precipitate the particles into the atmosphere (Ripoll et al., 2020, and references therein). The state of the belts at any instant in time is thus a balance between the numerous competing source and loss processes.

At the outset of strong geomagnetic disturbances and/or after the arrival of solar wind transient structures, both rapid source (e.g., in‐situ local acceleration via whistler‐mode chorus waves) and rapid loss (e.g., loss to the compressed magnetopause) processes become enhanced. These processes can dramatically alter the global configuration of the belts on timescales on the order of a few minutes or less. Outside of these rapid changes, the quiescent state of the belts is largely determined by two competing processes, inward radial transport, which acts as a source, and pitch‐angle scattering, which removes particles from the belts and precipitates them into the Earth's upper atmosphere. Both of these processes are usually described with a quasilinear Fokker‐Planck diffusion equation and are mediated by resonant wave‐particle interactions. Ultralow frequency waves, ∼mHz fluctuations in the inner magnetospheric electric and magnetic fields, are the predominant driver of the inward radial diffusion (Lejosne & Kollmann, 2020). In this work, we focus our attention on the slow, steady particle decays that are the hallmark signature of pitch‐angle diffusion.

In our previous work (Claudepierre et al., 2020b), we identified exponential decays in Van Allen Probe (Mauk et al., 2013) radiation belt electron flux measurements, from which we computed mean decay (e‐folding) timescales as a function of the McIlwain *L* parameter (*L* ∼ 1−6) and energy (∼30 keV to 4 MeV). In a companion paper (Claudepierre et al., 2020a), we compared the observed decay timescales with theoretical expectations for pitch‐angle diffusion from plasmaspheric hiss waves, ground‐based very‐low‐frequency (VLF) transmitter waves, electromagnetic ion cyclotron (EMIC) waves, and Coulomb collisions with neutral particles in the Earth's upper atmosphere and charged particles in the ionosphere. Good qualitative agreement was found between the observed decay timescales and our theoretical estimates. However, quantitative agreement was lacking in some portions of *L*‐energy space, particularly in the inner zone (*L* < 2.5) where calculated lifetimes from pitch‐angle diffusion were ∼1 order of magnitude larger than the observed decay timescales. Several known shortcomings in our treatment and approach were described in Claudepierre et al. (2020a), which we revisit in what follows.

The remainder of this paper is structured as follows. In Section 2, we describe the theory and methods that we use, along with the pitch‐angle scattering processes that are included in our theoretical calculations. In particular, we provide an updated treatment of scattering via VLF and low‐frequency (LF) transmitter waves based on recent work (Gu et al., 2021; Ma et al., 2022; Meredith et al., 2019). We also describe the explicit incorporation of lightning‐generated whistler (LGW) waves into our scattering calculations, which was ad‐hoc in our original treatment. We compare these revisions to our earlier work in Section 3.1, where we also provide a rough calculation of the effect that the drift loss cone has on electron decay timescales in the inner radiation belt region. In Section 3.2, we explore the sensitivity of the lifetime calculations to the choice of plasmaspheric density model. The importance of ionization energy loss, sometimes referred to as “Coulomb energy drag,” in producing loss in the inner belt has been emphasized recently by Albert et al. (2020) and we investigate this in Section 3.3. Here, 2D (pitch angle and momentum) Fokker‐Planck simulations are used as a tool for analysis. A brief discussion of the findings is presented in Section 4 and concluding remarks are given in Section 5.

## 2 Theory and Methods

### 2.1 1D Pitch‐Angle Diffusion

Pitch‐angle diffusion is described by the modified Fokker‐Planck equation (e.g., Lyons & Thorne, 1973):

(1) $$\frac{\partial f}{\partial t}=\frac{1}{G}\frac{\partial }{\partial \alpha }\left(D_{\alpha \alpha }G\frac{\partial f}{\partial \alpha }\right)$$

where *f* is the distribution function (phase space density), *α* is the equatorial pitch angle, and *D*_*αα*_ is the bounce‐averaged pitch‐angle diffusion coefficient. The Jacobian factor, *G*, for transforming from adiabatic invariant coordinates is given by *G* = *T*(*α*) sin(2*α*), where *T* ≈ 1.30–0.56 sin  *α* is a term that approximates the pitch‐angle dependence of the normalized bounce time along a dipole field line.

Under the assumption that the solution to Equation 1 is separable, that is, that *f*(*α*, *t*) = *g*(*α*)*h*(*t*), and that the time dependence follows exponential decay (*h*(*t*) ∼ exp(−*t*/*τ*)), we obtain a 1D ordinary differential equation (ODE) for the evolution in pitch angle:

(2) $$\frac{1}{G}\frac{d}{d\alpha }\left(D_{\alpha \alpha }G\frac{dg}{d\alpha }\right)=-\frac{1}{\tau }g$$

When considered over the pitch‐angle interval from the loss cone angle (*α*_*LC*_) up to 90° and formulated with the usual boundary conditions, for example:

(3) $$g=0\;\text{at}\;\alpha =\alpha_{LC}\;\text{and}\;\frac{dg}{d\alpha }=0\;\text{at}\;\alpha =\pi /2,$$

this second‐order linear ODE is of the “Sturm‐Liouville” type (Powers, 1999). The family of solutions is described in terms of eigenfunctions, *g*(*α*), and the associated eigenvalues, *λ* = 1/*τ*. Under modest continuity assumptions on the diffusion coefficient, *D*_*αα*_, Sturm‐Liouville theory guarantees that the eigenvalues (*λ*_1_, *λ*_2_, *λ*_3_, …) that correspond to each eigenfunction (*g*_1_, *g*_2_, *g*_3_, …) are real and ordered, such that *λ*_1_ < *λ*_2_ < *λ*_3_ < ⋯. Initially, the solution to Equation 1 will consist of the superposition of multiple different eigenmodes. However, once this initial transient behavior subsides, the long‐term evolution will be that of exponential decay of the lowest order eigenmode, *g*_1_(*α*), on the longest timescale *τ*_1_ = 1/*λ*_1_. In what follows, we will omit the subscript 1 and refer to this lowest‐order solution, *g*(*α*) = *g*_1_(*α*), uniquely as the “slowest decaying eigenmode” (SDE) of the pitch‐angle diffusion process, which decays with the e‐folding timescale *τ* = *τ*_1_. An approximate solution for this decay timescale of the SDE, or “lifetime,” is given by the explicit integral (Albert & Shprits, 2009):

(4) $$\tau \approx \int_{\alpha_{LC}}^{\pi /2}\frac{1}{2D_{\alpha \alpha }\tan\alpha }d\alpha$$

### 2.2 Pitch‐Angle Diffusion Coefficients

One of the primary goals of this work is to consider several mechanisms that produce quasilinear pitch‐angle diffusion, calculate the decay timescales associated with each process, and compare the results with observed decay timescales. Solving for the decay timescale, *τ*, from either Equation 2 (subject to the indicated boundary conditions) or Equation 4, requires the specification of the pitch‐angle diffusion coefficient, *D*_*αα*_.

We obtain these coefficients in the usual manner following the methods described in our previous work (Claudepierre et al., 2020a). Briefly, we use the Full Diffusion Code (Ni et al., 2008) to calculate the bounce‐and‐drift‐averaged diffusion coefficients in a dipole field using the plasma density model of Ozhogin et al. (2012) at *L* < 4. For the scatterings due to wave‐particle interactions, we specify the amplitudes, frequency spectra, and wave normal angle spectra from various empirical wave models (see below). The magnetic‐latitudinal range for the interactions is assumed to be ±45° for hiss and EMIC waves, and from the equator to the altitude of 800 km for the transmitter and LGW waves. Resonant harmonics between ±10 are considered and the calculations are performed on a grid in *L* from 1 to 4 (Δ*L* = 0.1), energy from 0.1 keV to 10 MeV (in 71 logarithmically spaced channels), and equatorial pitch angle from 1° to 89.5° (Δ*α* = 2°). For the scatterings due to Coulomb collisions, we follow the methodology of Abel and Thorne (1998), obtaining the atmospheric neutral species (N_2_, O_2_, Ar, He, O, H, and N) from the MSIS90 empirical model (Hedin, 1991) and the charged species (e^−^, NO^+^, O^+^, $O_{2}^{+}$, H^+^, He^+^, and N^+^) from the IRI2016 model (Bilitza et al., 2017).

There are several wave modes that are known to be important for scattering electrons in pitch angle in the inner radiation belt and slot region (*L* < 4). Hiss is a broadband (*f* ≈ 100 Hz to 1 kHz), incoherent whistler mode wave that occurs primarily in the high‐density plasmasphere. Wave amplitudes vary with geomagnetic activity, with typical values in the 10–100 pT range, and are most intense on the dayside. LGW waves are waves injected into the *L* < 4 region from the troposphere following lightning strikes. The waves reflect within the plasmaspheric cavity and eventually migrate to a preferred *L*‐shell region dictated by the local lower‐hybrid resonance frequency. LGW waves are typically discrete, impulsive events with wave frequencies on the order of a few kHz and amplitudes in the ∼1–10 pT range. VLF transmitter waves are whistler mode waves that are injected into the *L* < 3 region from high‐powered, ground‐based radio wave transmitters. These waves, with amplitudes of several pT, are essentially monochromatic and propagate at the transmitting frequency of the station (typically ∼15–25 kHz). Both LGW and VLF transmitter waves have a strong asymmetry in magnetic local time (MLT), with more intense amplitudes on the nightside due to collisional damping in the D‐region ionosphere.

### 2.3 Empirical Wave and Scattering Models

Table 1 organizes the scattering models and calculation characteristics that we will use in the present study. For example, “lifetime model 0” (LM0) represents the empirical wave models and assumptions that were used in Claudepierre et al. (2020a). Each subsequent row in the table corresponds to a different lifetime model that we will consider, making progressive refinements to the baseline model, LM0. The second column, “*τ* Method,” indicates whether the approximate formula (Equation 4) is used to compute the lifetime, or whether the exact solution is obtained. The exact calculation is performed by solving the 1D ODE (Equation 2) for *τ* and the equilibrium eigenfunction, *g*, via a shooting method (e.g., Albert, 1994; Albert et al., 2020). The third column in the table, “*τ* LC Assumption,” indicates whether the dipole field loss cone (LC) angle is used when calculating *τ* from Equation 2 or Equation 4, or whether the drift‐loss cone angle (DLC) from the International Geomagnetic Reference Field (IGRF; Alken et al. (2021)) model is used. The subsequent columns in Table 1 (Hiss, LGW, …) denote the scattering models as either “Model A,” our original baseline empirical wave and Coulomb models from Claudepierre et al. (2020a), or “Model B,” which represent refinements of each Model A.

**Table 1:** Summary of Wave Models and Scattering Calculations Used to Define Each Lifetime Model

Lifetime	*τ*	*τ* LC	Hiss	LGW	VLF	Coulomb	EMIC
model (LM)	method	assumption	model	model	model	model	model
LM0^a^	Approx.^b^	Dipole	A	n/a^d^	A	A	A
LM1	Exact^c^	Dipole	A	n/a^d^	A	A	A
LM2	Exact^c^	Dipole	A	n/a^d^	B	A	A
LM3	Exact^c^	Dipole	B	B	B	A	A
LM4	Exact^c^	DLC/IGRF	B	B	B	B	A

^a^ The setup used in Claudepierre et al. (2020a).

^b^ Calculated from Equation 4.

^c^ Obtained via shooting method on Equation 2.

^d^ Ad‐hoc incorporation into hiss model A (see text).

Hiss model A is defined using the statistical wave frequency spectrum obtained by Li et al. (2015), along with the statistical amplitudes and their dependence on *Kp* from Spasojevic et al. (2015), and the wave normal angle spectrum from Ni et al. (2013). In Claudepierre et al. (2020b), we extrapolated the hiss spectrum from 4 to 7 kHz as an approximate way to incorporate LGW waves into our calculations. Thus, there is no model A for LGW in Table 1. Model B for LGW waves uses the statistical wave database of Green et al. (2020), who parameterized LGW waves from Van Allen Probe measurements at *L* < 4, carefully distinguishing them from hiss waves in the overlapping frequency range. Hiss model B is identical to hiss model A, except that the extrapolation of the spectrum from 4 to 7 kHz has been removed.

VLF model B represents a reformulation of the empirical VLF model A (Ma et al., 2017) and is described in greater detail in Ma et al. (2022). The most notable differences are that the statistical database was extended in time from 2016 to the end of the Van Allen Probes mission in 2019, that the dependence on geomagnetic activity was removed, and that the frequency range was extended from 30 kHz up to 200 kHz to account for non‐negligible transmitter wave power observed at these higher frequencies. In addition, both the wave normal angle variation with latitude and the power ratio between ducted and unducted wave intensity were changed to follow recent work from Gu et al. (2021). This study found that unducted propagation dominates over ducted propagation in both the occurrence and intensity of the waves.

In the present study, the EMIC model is not changed from that used in Claudepierre et al. (2020b) and is only mentioned in passing since the focus of this work is on loss timescales at *L* < 4. In this region in the empirical wave model, the EMIC wave amplitudes are small and only reach appreciable levels for high geomagnetic activity. In addition, the EMIC waves from the empirical model mainly affect higher energy electrons than the energy range (30 keV to 4 MeV) under consideration in this study.

**Figure 1:**
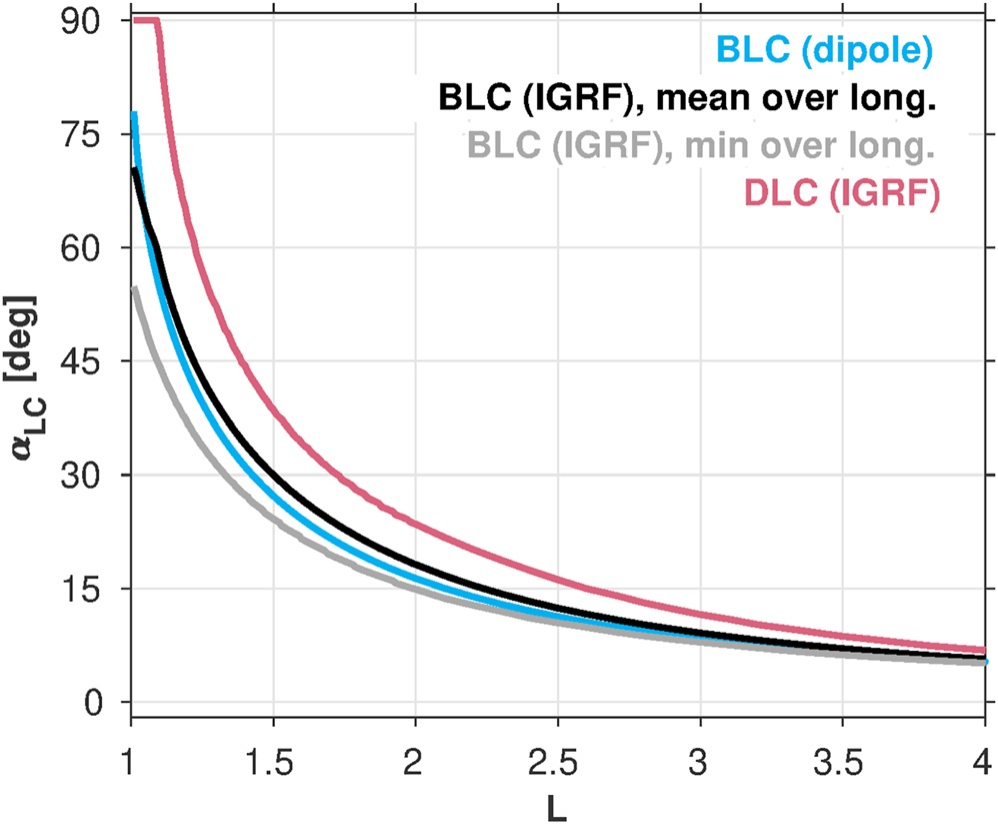
A comparison of the equatorial loss cone angles obtained from two magnetic field models: A dipole model and the IGRF model. The cyan curve shows the bounce loss cone (BLC) angle in a dipole field. The black curve shows the BLC angles from the IGRF model averaged over all longitudes. The gray curve shows the minimum of the BLC angles from the IGRF model over all longitudes. The red curve shows the maximum of the BLC angles from the IGRF model over all longitudes, that is, the drift loss cone (DLC) angle.

Finally, Coulomb model B is identical to Coulomb model A, except that the IGRF DLC angle is used instead of the dipole loss cone angle in the diffusion coefficient calculations. The bounce loss cone pitch angle is determined under the assumption that the electrons are lost at 100 km altitude using a dipole magnetic field model. The drift loss cone pitch angle is determined by computing the 100‐km bounce loss cone angle at different longitudes from the IGRF model and selecting the largest of these pitch angles. Figure 1 compares these two angles (in red and cyan curves, respectively) indicating that there can be significant differences at *L* ≲ 2.5. Thus, we anticipate that this should have an effect on the lifetimes in this region.

For the dipole field model (Coulomb model A), our methods to calculate diffusion coefficients and Coulomb scattering are very similar to those of Albert et al. (2020) and Selesnick (2016) except for the ionospheric model used. For the IGRF model, the Coulomb scattering rates are calculated for field lines at different longitudes and then averaged to obtain the drift‐averaged diffusion coefficients. After the diffusion coefficients are calculated, the loss cone pitch angles (either bounce or drift) are used to calculate the electron lifetimes. We note that, although the magnetic field lines at different longitudes in the IGRF model are considered in Coulomb model B, the neutral and charged particle density profiles are the same between Coulomb models A and B, because each field line in the IGRF model will pass through different MLTs over time.

We emphasize that the wave scattering models (hiss, LGW, VLF, and EMIC) are not changed when the DLC angle is used in place of the dipole angle since, when computing the diffusion coefficients, the choice of loss cone angle is only relevant for Coulomb scattering. Thus, there is no model C for hiss, LGW, VLF, or EMIC; model B can be used when the DLC effects are considered below.

## 3 Results

In this section, we begin by comparing the observed lifetimes with the various revisions to our lifetime models under 1D pitch angle diffusion. In Section 3.2, we examine the role of the plasmaspheric density in controlling the lifetimes, and we compare our lifetimes with Albert et al. (2020), who present similar lifetime calculations but treat LGW and VLF waves using a different approach. Finally, in Section 3.3, we analyze the Coulomb energy drag process using 2D simulations.

### 3.1 Comparison of the Different Lifetime Models Under 1D Pitch Angle Diffusion

**Figure 2:**
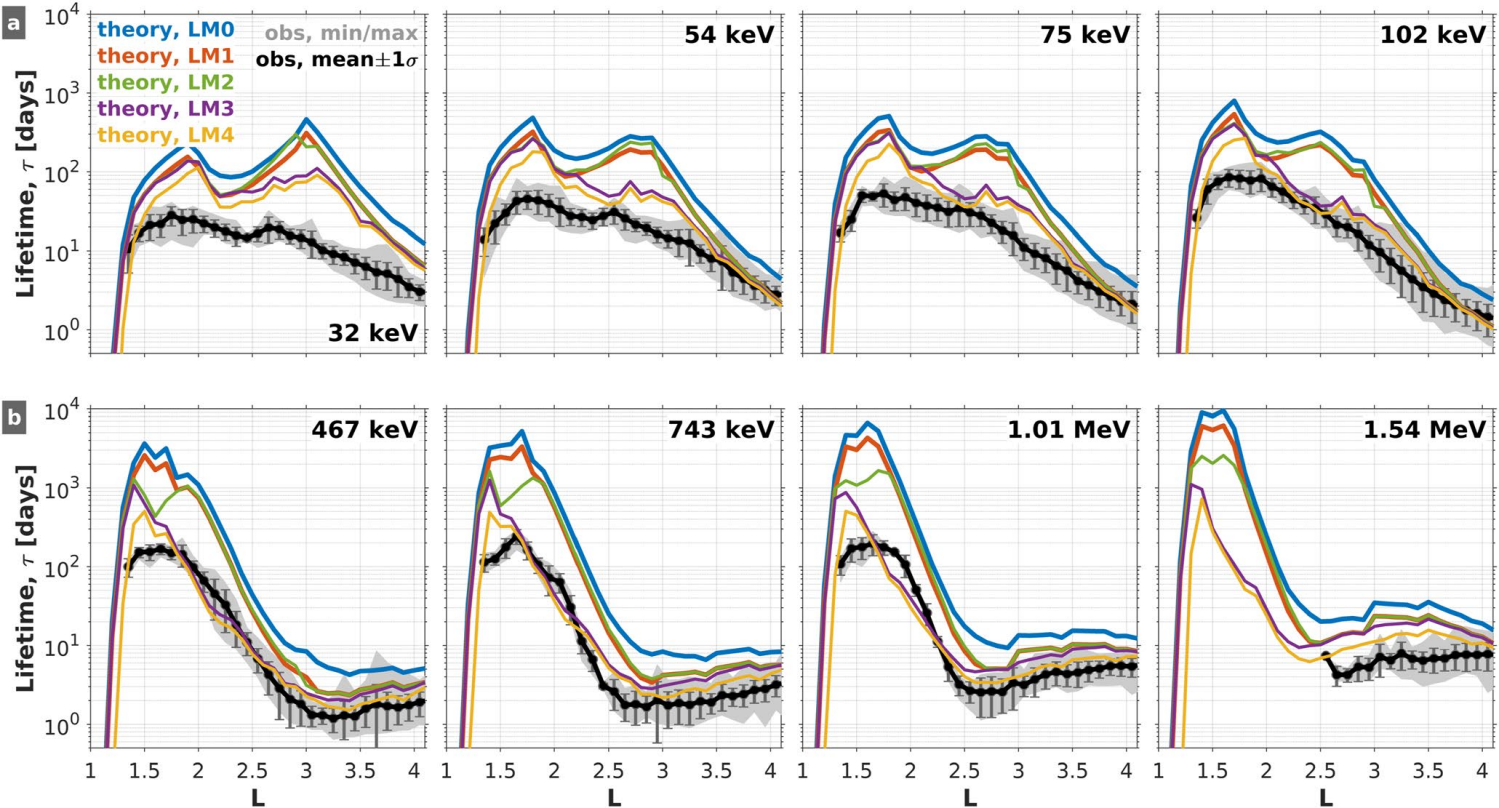
Comparison of observed decay timescales (black/gray) with theoretical calculations (colors) for *Kp* = 0 and 5 different lifetime models (LMs; see Table 1). Each panel shows the lifetime profiles versus *L* at a fixed energy, and the five LMs are summarized as follows: LM0: Claudepierre et al. (2020a); LM1: Exact lifetime calculation (shooting method); LM2: Revision of VLF transmitter scattering; LM3: Explicit inclusion of scattering due to LGW waves; and LM4: Use of the IGRF drift‐loss cone angle when computing the lifetime.

Figure 2 compares the observed decay timescales obtained by Claudepierre et al. (2020b) with those calculated from the theoretical lifetime models (LMs) described above (i.e., Table 1). Before we make the comparisons, we remark on a couple apparent differences between the results present in Figure 2 and those presented in Claudepierre et al. (2020b). First, we note that the curves from the five LMs are less smooth than those shown in Claudepierre et al. (2020a) because in our previous work we interpolated the theoretical lifetimes to the observed energy and *L* bins. In the present study, there is no interpolation and the nearest energy bins are used (the *L* resolution is the same, 0.1 *L*). The energy channel labels shown in the figure are taken from the observations, but the channels that were used in the diffusion coefficient/lifetime calculations are quite close, typically within <5% of the observed channel. Aside from these distinctions, the blue curve for LM0 and the observed lifetimes shown in Figure 2 are the same as presented in Claudepierre et al. (2020a).

As described in Claudepierre et al. (2020b), the qualitative trends in Figure 2 are consistent between the theoretical calculation using LM0 and the observed lifetimes. For example, the longest lifetimes are found in the inner zone at *L* < 2, and the lifetimes generally decrease with increasing *L* at *L* > 2. At fixed *L* in the inner zone, say *L* = 1.5, both the theoretical lifetimes from LM0 and the observed lifetimes increase with increasing energy. Similarly, in the slot region at fixed *L*, say *L* = 3, both the theoretical and observed lifetimes display a local minimum near 500 keV. In contrast to this good qualitative agreement, the quantitative agreement between LM0 and the observed decay timescales is poor in many regions of *L*‐energy space, where order‐of‐magnitude (or greater) differences are noted at *L* < 3 across all energies.

As a first iteration on our baseline calculation, LM0, we highlight LM1 as the orange curves in Figure 2. In LM1, the lifetimes are calculated exactly via a shooting method on Equation 2, rather than with the approximate formula (Equation 4) that was used for the calculations in LM0. Comparing LM0 with LM1, we see an overall reduction in lifetimes by a factor of ∼2 across all *L* and energy (*E*) bins. (Note that in some *L*/*E* bins, the orange curve is not always distinguishable from other curves that may overlap it.) This indicates that the approximate formula derived by Albert and Shprits (2009) results in lifetimes larger than the exact calculation by a factor of ∼2×, on average. In all subsequent calculations, we use the exact formulation to compute lifetimes from the pitch‐angle diffusion coefficients.

As an iteration on LM1, we now consider the revisions to our VLF transmitter wave empirical model described in Section 2.3. Comparing the theoretical lifetimes from LM1 (orange curves) with this iteration, LM2 (green curves), reveals that the impact of the revisions is minimal, especially at lower energy (*E* < 100 keV; panel (a)). The only appreciable differences are at higher energies (panel (b)) between *L* ≈ [1.3, 1.8], where the lifetimes in LM2 are reduced relative to LM1.

This reduction in electron lifetimes is mainly due to the more accurate wave normal angle distributions, and partly due to the inclusion of the LF (30–200 kHz) transmitter waves. We consider the *L* shell and latitude dependencies of wave normal angles for unducted transmitter waves in LM2 based on raytracing results (Gu et al., 2021; Ma et al., 2022). The LF transmitter waves could resonate with electrons at lower energies or higher pitch angles than the waves at frequencies below 30 kHz, although the LF transmitter wave power is much weaker.

More pronounced changes are seen when comparing LM2 (green curves) with LM3 (purple curves), where the LGW waves are explicitly incorporated into the scattering calculations. For example, at 102 keV, we see that the LGW waves reduce the lifetimes by nearly an order of magnitude at *L* ≈ 2.5 and generally reduce the lifetimes across a broad spatial region from *L* ≈ 2 to 3.5. Similar lifetime reductions relative to LM2 are seen at both lower and higher energies, with the spatial region of influence moving progressively earthward with increasing energy. This is in accordance with the roughly *L* ^−6^ scaling of the minimum energy expected for cyclotron resonance with whistler mode waves for the magnetic field and plasma density models used here (Claudepierre et al., 2020a; Ma et al., 2016; Mourenas et al., 2012).

The incorporation of the LGW waves into LM3 produces another interesting effect relative to LM0. As described in Claudepierre et al. (2020a), the local minimum in the LM0 lifetimes near *L* ≈ 2 at the lower energies (panel (a)) is due to scattering from the VLF transmitter waves, which produces a bifurcation in the inner belt (see also Hua et al. (2020)). When the LGW waves are explicitly included in LM3, the second local maxima in the lifetimes (the one at higher *L*) is reduced, so that the “valley” produced by the local minimum is less pronounced in LM3 relative to LM0. This leads to a better agreement between LM3 and the observed lifetimes, where the local minimum due to VLF wave scattering is observed but is less pronounced than in LM0. In general, the inclusion of the electron scattering from LGW waves has the largest impact of the effects considered in Figure 2 and brings our theoretical calculations into better agreement with the observed decay timescales.

As a final iteration, we consider the influence that the drift loss cone can have on the decay timescales in the low *L* region (i.e., Figure 1). By definition, an electron that is pitch‐angle scattered into the bounce loss cone will be lost from the belts in one‐quarter bounce time, whereas an electron that is scattered into the drift loss cone will be lost within one drift period. Since we are considering drift‐and‐bounce‐averaged electron dynamics and decays that occur over multi‐day timescales, the drift loss cone angle is the more relevant loss cone angle for scattering losses (i.e., the electron drift periods are much less than the multi‐day timescales in question).

**Figure 3:**
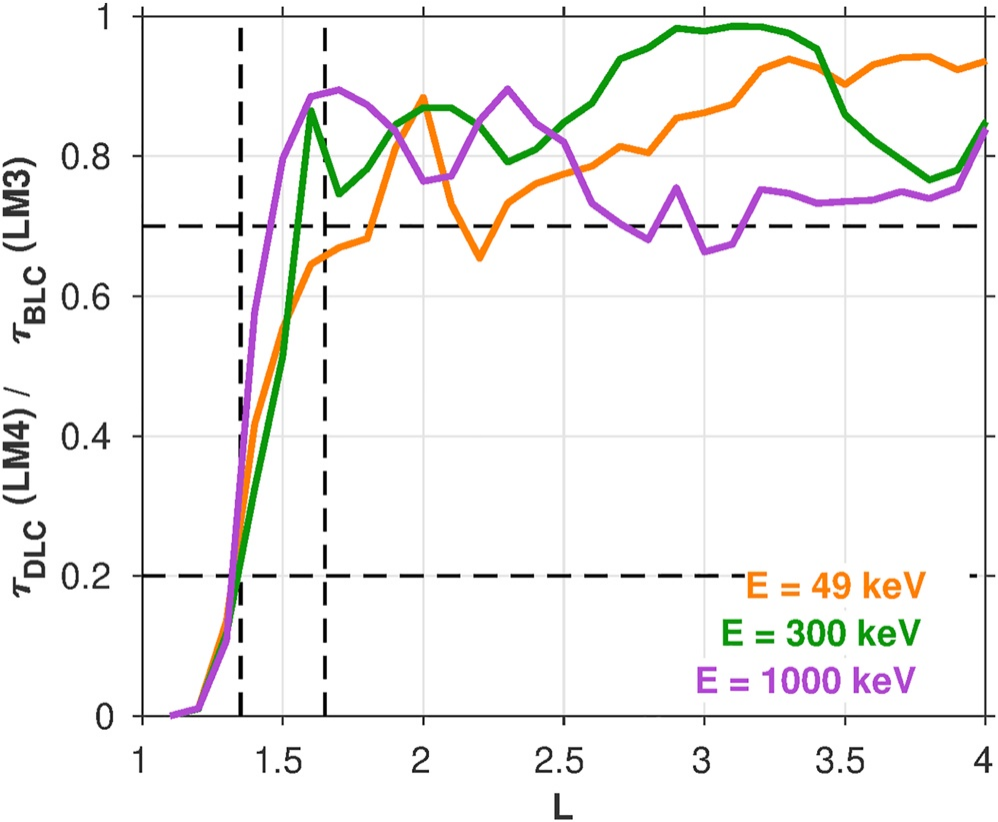
A comparison of lifetime ratios versus *L* using the lifetimes from LM3 (dipole/BLC) and LM4 (IGRF/DLC) for three representative energies (50 keV, 300 keV, and 1 MeV).

Lifetime model 4 (LM4) in Figure 2 shows the effect of using the IGRF drift loss cone angle in place of the dipole loss cone angle when computing the lifetimes. While the exact shooting calculation is used here, a consideration of the integral in Equation 4 immediately illustrates the effect: Using the DLC angle in place of the BLC angle in the integration limits will result in reduced lifetimes. Indeed, this is borne out in the calculated lifetimes shown in Figure 2. To examine this more closely, in Figure 3, we compare the lifetime ratios between LM3 and LM4 at three representative energies. The largest differences are at *L* ≲ 1.7, since this is where the BLC and DLC angles differ most significantly. Here, the lifetimes are reduced in LM4 relative to LM3 by a factor of ∼2–5 at *L* = [1.4, 1.6], and by an order of magnitude or more at *L* < 1.3. At higher *L* > 1.7, the lifetimes are reduced by a smaller amount, ∼20%. The increase of the loss cone size when changing from the BLC to the DLC contributes to the reduction of these lifetimes. At low *L* shells (*L* ≤ 1.3), the minimum cyclotron resonance energy due to VLF transmitter waves is higher than ∼1 MeV, and the small pitch angle diffusion coefficients outside the BLC in the ∼100 keV to several MeV energy range have a large influence on the lifetimes (e.g., Equation 4). Increasing the loss cone pitch angle from the BLC to the DLC reduces the regime of the small pitch angle scattering rates, and significantly reduces the electron lifetimes at *L* ≤ 1.3.

It is interesting to note that while the differences between LM3 and LM4 trend with *L* as one might expect based on the differences between the DLC and BLC angles shown in Figure 1, this behavior is not seen consistently across all energies shown. For example, comparing LM3 and LM4 at 102 keV in Figure 2, there is little difference in the lifetimes at *L* > 3, while at 1.54 MeV, there are clear differences at *L* > 3. While the only distinction between LM3 and LM4 is the choice of loss cone angle, there are other more subtle factors that may lead to this peculiarity. For example, the relative effectiveness of the various scattering mechanisms (e.g., hiss vs. LGW) near the loss cone is different at different energies.

It is clear that the lifetimes obtained from LM3 and LM4 represent an improvement on LM0, as we have obtained better quantitative agreement with the observations. Thus, we proceed with LM3 as our new “baseline” model for further analysis and comparisons. While the effect of the drift loss cone demonstrated in LM4 is important, particularly at *L* < 1.5, LM3 is most readily compared with previous works in this area since nearly all such efforts use the dipole‐field loss cone angle when computing lifetimes. It is also important to acknowledge and emphasize that the improved agreement demonstrated between the observed decay timescales and those from LM3 should not be interpreted to mean that LGW waves are more important than the other scattering mechanisms in the *L* < 4 region. Their impact is obvious in Figure 2 relative to the other effects considered because LGW waves were absent from our earlier work.

### 3.2 Lifetime Sensitivity to Plasma Density and Comparisons With Albert et al. (2020)

Albert et al. (2020) and Starks et al. (2020) have taken a different approach to analyze the role of LGW and VLF transmitter waves in inner zone lifetimes. Rather than use statistically averaged empirical wave models, as we have done here, they model the waves from their ground sources to 660 km altitude using a full‐wave code, and then use raytracing to propagate the waves into the *L* < 4 region. Given these contrasting techniques, it is instructive to compare the theoretical lifetimes from our approach with theirs, all relative to the observed decay timescales.

**Figure 4:**
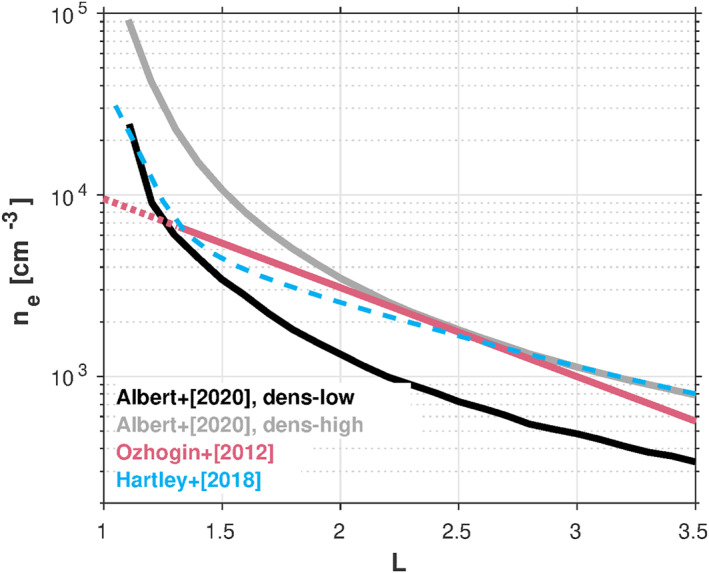
Comparison of the electron density model used in this study (Ozhogin et al., 2012; red curve) with those used in Albert et al. (2020) (black/gray curves) and the hiss‐inferred values from the empirical model of Hartley et al. (2018). The dashed portion of the Ozhogin et al. (2012) profile is an extrapolation of the model below its region of validity (to altitudes <2,000 km). The Hartley et al. (2018) curve is the median over all magnetic latitudes (their fig. 6d).

The Albert et al./Starks et al. calculation provides profiles of the LGW and VLF wave electric and magnetic fields organized by *L*, from which they compute diffusion coefficients using the single‐wave formulation of Albert (2010). For the plasmaspheric density model, they use a relatively full (dens‐high) and a relatively empty version (dens‐low) of the diffusive equilibrium model of Angerami and Thomas (1964). Figure 4 compares these two density models with the one used in this study (Ozhogin et al., 2012), alongside the Hartley et al. (2018) model, which was constructed using plasmaspheric hiss measurements. Since the prevalence of ducting due to field‐aligned plasmaspheric density enhancements/depletions is not well constrained (Gu et al., 2021), Albert et al. (2020) calculate ducted solutions by setting the wave normal angle to 0° and restricting to strict parallel propagation (unducted solutions are obtained without any restrictions on the propagation). Their models of hiss and Coulomb scattering are similar to what we have used.

**Figure 5:**
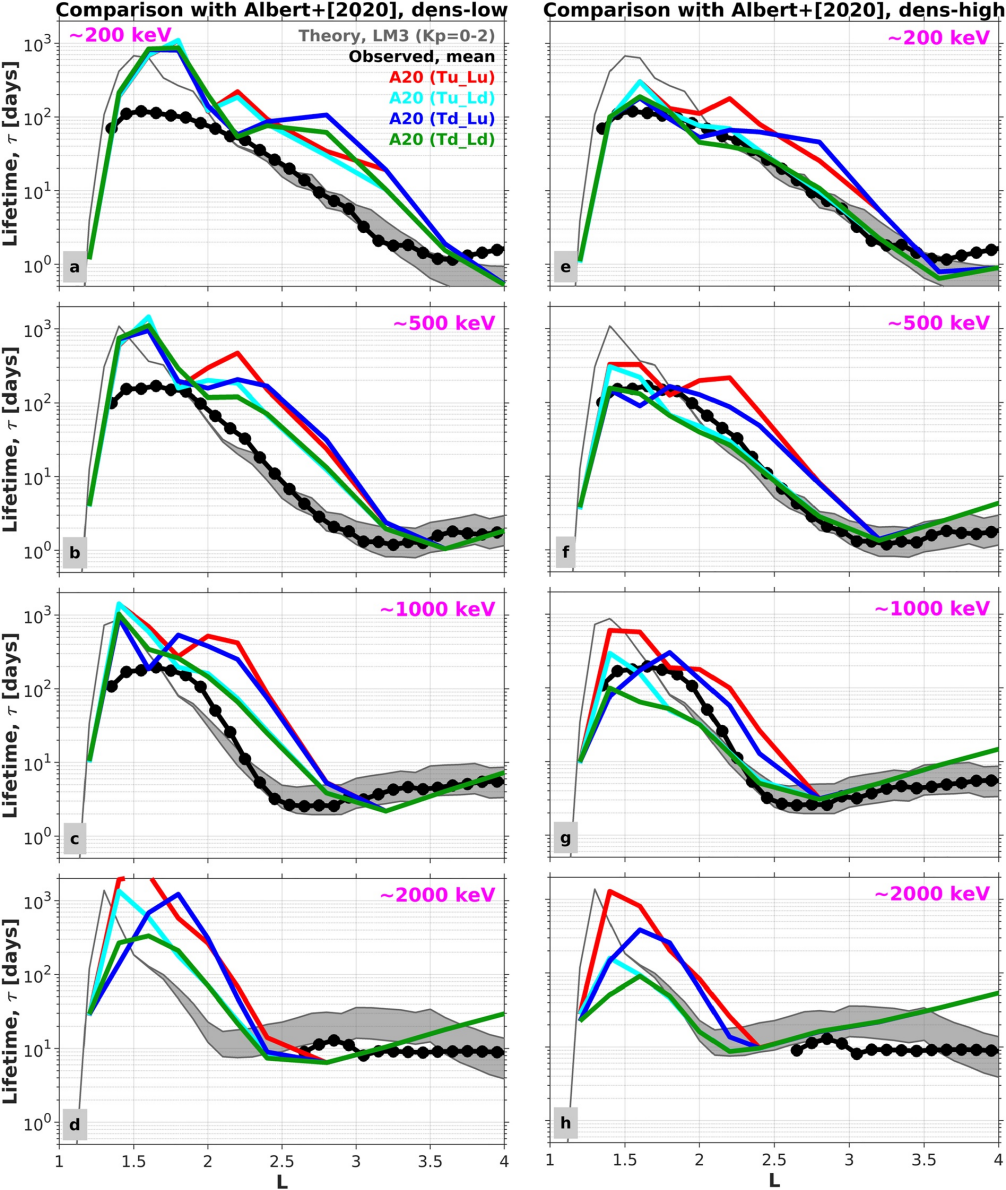
Comparisons with the lifetimes calculated in Albert et al. (2020) for their low plasmaspheric density model (“dens‐low,” (a)–(d)) and their high‐density model (“dens‐high,” (e)–(h)) at four different energies plotted. The Albert et al. calculations are shown as colored curves with each of the four curves representing different combinations of ducted (d) and unducted (u) propagation for LGW (L) and VLF (T) waves. The observed lifetimes are shown with black circular symbols and our lifetime model 3 (LM3) is shown in gray with the shaded region indicating the range of lifetimes for different activity levels, *Kp* = 0–2.

Figure 5 compares the lifetimes obtained by Albert et al. (2020) (henceforth, “A20”) with our lifetime model 3 (LM3). For the A20 lifetimes obtained using their dens‐low plasmasphere model (left column, panels (a)‐(d)), we see that our theoretical calculations are similar to theirs below *L* = 1.5, in terms of both the maximum lifetime and the shape of the profile in *L*. Note that the theoretical lifetimes calculated in this region disagree significantly with the observed values, larger by factors ∼5–10 for both LM3 and A20. As *L* increases, we see that the agreement between our theoretical calculations and A20 begins to diverge, with the A20 values larger than our computed lifetimes in the *L* = 2–3 region. This disparity may be due to combination of effects, such as the differences in how the LGW and VLF waves are treated and/or the use of different plasmaspheric models. For example, larger electron densities produce smaller lifetimes, all other effects being equal, and we see that the electron densities from the dens‐low model are somewhat lower than those from the Ozhogin et al. (2012) model at *L* ≳ 1.5 (Figure 4). This may lead to larger lifetimes from the A20 calculations relative to ours in this region. At the higher *L* values (*L* > 3), where the hiss wave scattering begins to be the dominant scattering mechanism, the A20 lifetimes generally agree with ours since both approaches use the same hiss model. We emphasize that the boundaries of the *L* regions described here (i.e., *L* < 1.5, *L* = 2 − 3, and *L* > 3) are notional and in reality are energy‐dependent, due to the *L* ^−6^ dependence to the cyclotron resonance condition noted above.

Comparing the left and right columns in Figure 5 illustrates the sensitivity of the theoretical lifetime calculations to the assumed electron density model. First, above *L* = 1.5, note that the densities from the dens‐low and dens‐high models only differ from one another by a factor of ∼2–3 (Figure 4), yet the A20 lifetimes can differ by factors on the order of 5–10 depending on *L*. In particular, we see that at *L* < 1.5, where our LM3 disagrees significantly with the observed lifetimes, the A20 dens‐high lifetimes are in much better agreement with the observed values (Figure 5, right column). It is clear that this improved agreement over dens‐low (and LM3) is solely due to the choice of the density model, since this is the only thing that is different between the left and the right columns in Figure 5.

We emphasize that at *L* < 1.5, the electron densities from the dens‐high model are considerably larger than the other density models shown in Figure 4. In particular, dens‐high begins to diverge from the Ozhogin et al. (2012) empirical model near *L* = 2 and is similarly inconsistent with the model of Hartley et al. (2018) at *L* < 2. At *L* < 1.5, we see that the hiss‐inferred densities from Hartley et al. (2018) are significantly lower than dens‐high and are in much better agreement with dens‐low. We thus argue that the dens‐high model densities may be unrealistically large at *L* < 1.5 and could lead to inaccurate lifetime calculations in this region. While the dens‐high versus dens‐low differences could potentially be appropriate for accounting for day/night asymmetries in the density at low *L* due to the ionosphere, existing experimental evidence cannot confirm such a paradigm. Reliable electron density measurements are sparse in this region and there are very few data sources with which to compare. Future work using new observations will be necessary to fully characterize the appropriateness of the dens‐high model at *L* < 2. Assuming that the dens‐high plasmaspheric density model is indeed inaccurate at *L* < 2, we thus seek an alternative mechanism to reconcile the disagreement between the observed lifetimes near *L* = 1.5 and those calculated from theory.

### 3.3 Coulomb Energy Drag Effects

In addition to their physics‐based approach for modeling VLF and LGW wave propagation and scattering, Albert et al. (2020) also examined how ionization energy loss influences electron lifetimes at low *L*. This consideration necessarily requires the reformulation of the problem from pure (1D) pitch angle diffusion into a 2D diffusion equation in momentum and pitch angle, along with a term that models the Coulomb drag process. Following Albert et al. (2020), we write this equation as:

(5) $$\frac{\partial f}{\partial t}=\frac{1}{G}\frac{\partial }{\partial \alpha }\left[G\left(D_{\alpha \alpha }\frac{\partial f}{\partial \alpha }+D_{\alpha p}\frac{\partial f}{\partial p}\right)\right]+\frac{1}{G}\frac{\partial }{\partial p}\left[G\left(D_{\alpha p}\frac{\partial f}{\partial \alpha }+D_{pp}\frac{\partial f}{\partial p}\right)\right]+\frac{1}{\gamma p}\frac{\partial }{\partial E}\left[\gamma p\frac{dE}{dt}f\right]$$

where *p* is the relativistic momentum, *γ* is the relativistic factor, *E* is the electron kinetic energy, and all other variables have been previously defined. We note that the Jacobian factor, *G*, for the coordinate transformation in this equation is different from the one in the 1D pitch angle diffusion equation (Equation 1) by a factor of *p* ^2^ and its definition is omitted here for brevity. The Coulomb energy drag rate is first calculated at different longitudes in the IGRF magnetic field model following the method in Albert et al. (2020). Then, the Coulomb energy drag, *dE*/*dt*, is obtained as the average of the drag rate over all longitudes.

To solve Equation 5, we follow the computational approach of Albert et al. (2020), using the same initial and boundary conditions, which were inferred from Van Allen Probe‐measured energy spectra and angular distributions. The bounce‐and‐drift averaged momentum (*D*_*pp*_) and mixed (*D*_*αp*_) diffusion coefficients are calculated in the same manner as described above for our pitch angle diffusion coefficients, *D*_*αα*_. These coefficients are specified using lifetime model 3 (LM3) and we conduct a separate simulation at four different *L* values: 1.6, 2.0, 2.4, and 3.1. The simulations are conducted using an energy grid with 151 logarithmically space values between 10 keV and 10 MeV, a pitch‐angle grid with resolution of 1°, and a simulation time step of 30 s. The electron phase space densities are assumed to be constant at the lower‐ and upper‐energy boundaries. The pitch angle boundary conditions are $D_{\alpha \alpha }\frac{\partial f}{\partial \alpha }+D_{\alpha p}\frac{\partial f}{\partial p}=0$ at *α* = 90°, and *f* = 0 inside the loss cone. The simulation is performed for 4,000 days, which is longer than the electron lifetimes of interest in this study.

To investigate the decay timescales associated with the combined effects of quasilinear diffusion and Coulomb energy drag, we carry out simulations as described above. Unlike in the 1D pitch angle diffusion case analyzed in Sections 3.1 and 3.2, the long‐term particle dynamics described by Equation 5 are not that of exponential decay in a single eigenmode. Thus, we must use a different approach to define and characterize decay timescales the in the simulated fluxes, in order to make meaningful comparisons with the observed timescales.

#### 3.3.1 Calculating the Decay Timescales From the 1D Simulations

**Figure 6:**
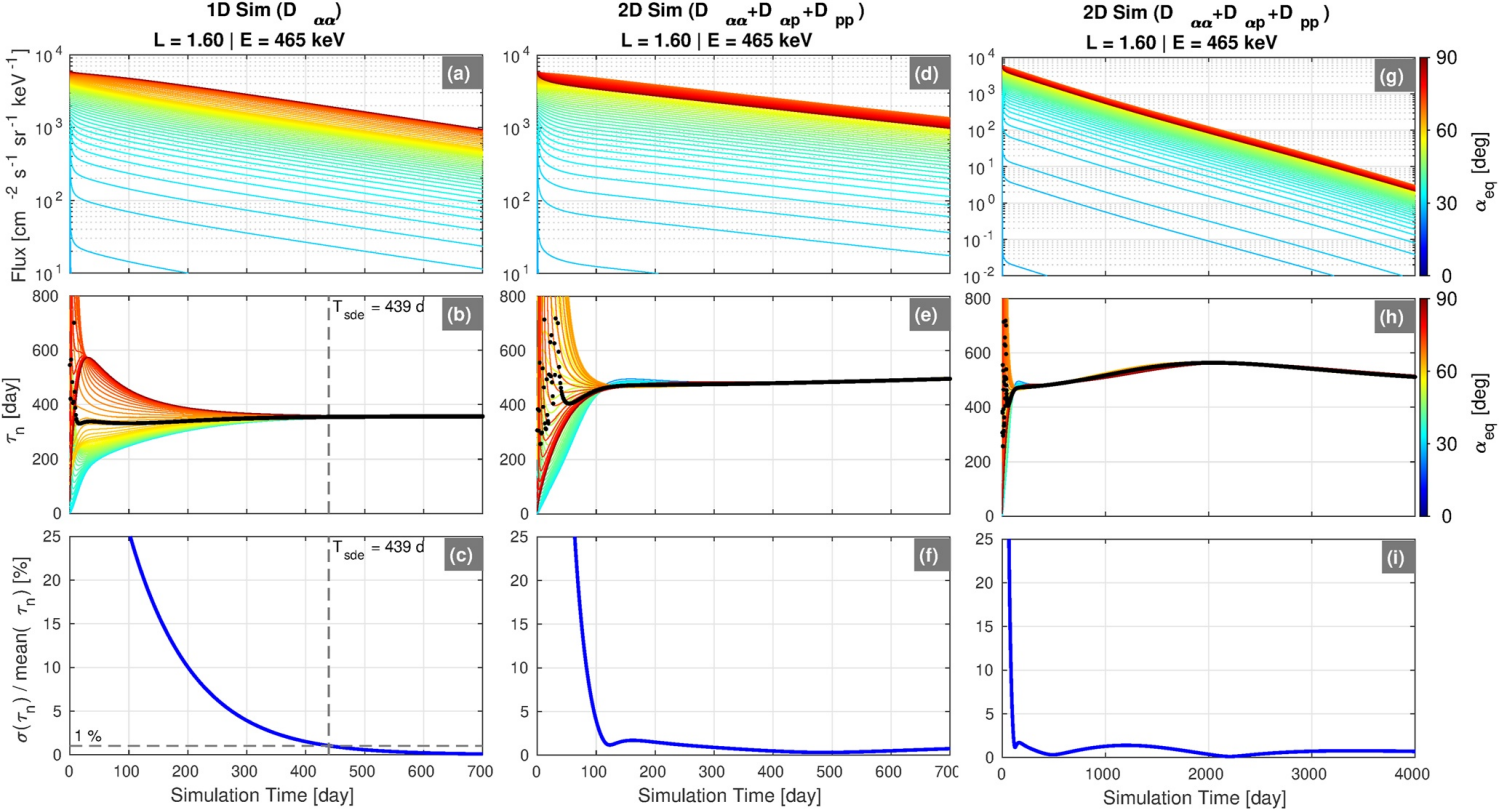
Summary of the results from the 1D ((a)–(c)) and 2D ((d)–(i)) simulations for 465 keV electrons at *L* = 1.6. The top row shows the simulated flux plotted against time with different colored curves for each equatorial pitch angle ((a), (d), and (g)). The middle row shows the decay timescale, *τ*_*n*_, at each time step, *n*, for all pitch angles ((b), (e), and (h)). The mean of *τ*_*n*_ over pitch angle is shown in black. The bottom row shows the mean relative error in *τ*_*n*_ expressed as a percentage ((c), (f), and (i)). This error is defined as the standard deviation of *τ*_*n*_ (*σ*(*τ*_*n*_)) divided by the mean of *τ*_*n*_ over all pitch angles, with the 1% error level indicated in panel (c). In panels (b) and (c), the time in the simulation when the slowest decaying eigenmode has been reached, *T*_*sde*_, is indicated. The right column is simply an expanded view of the time range shown in the middle column.

In the 1D pitch angle diffusion case, obtaining this decay timescale is well‐defined and straightforward since, by definition, the phase space density will eventually settle into the slowest‐decaying eigenmode. Figure 6a shows the solution to Equation 5 with only the pitch angle diffusion term (*D*_*αα*_) retained. The simulated phase space density is converted to flux (=*fp* ^2^) and is plotted for the first 700 days of the 4,000 days simulation for 465 keV electrons at *L* = 1.6. Aside from the transient behavior at the beginning of the simulation, it is clear that the fluxes are decaying exponentially at all pitch angles over the time interval shown.

We can calculate timescale of the slowest‐decaying eigenmode from the simulation as follows. Following Ni et al. (2013), we define the decay timescale, *τ*_*n*_, at each time step, *t*_*n*_, of the simulation as:

(6) $$\tau_{n}=-\frac{t_{n+1}-t_{n}}{\ln\left[j_{n+1}\left(\alpha \right)\right]-\ln\left[j_{n}\left(\alpha \right)\right]}\text{for}n=1,2,\ldots ,4000$$

where *j*_*n*_ is the simulated flux at time *t*_*n*_. Panel (b) shows this quantity plotted versus simulation time for all equatorial pitch angles. Initially, different pitch angles are decaying at different rates over a wide range of timescales, ∼200–600 days. Around day 300 in the simulation, we see that the decay timescales at each pitch angle start to converge to the single value of *τ*_*n*_ ≈ 380 days. We can determine the time it takes to reach this equilibrium state quantitatively by using the mean relative error of the *τ*_*n*_ values, which is plotted in panel (c). This quantity reaches the 1% level at day 439 in the simulation, which we define as “*T*_*sde*_,” the time in the simulation when the slowest decaying eigenmode has been reached.

**Figure 7:**
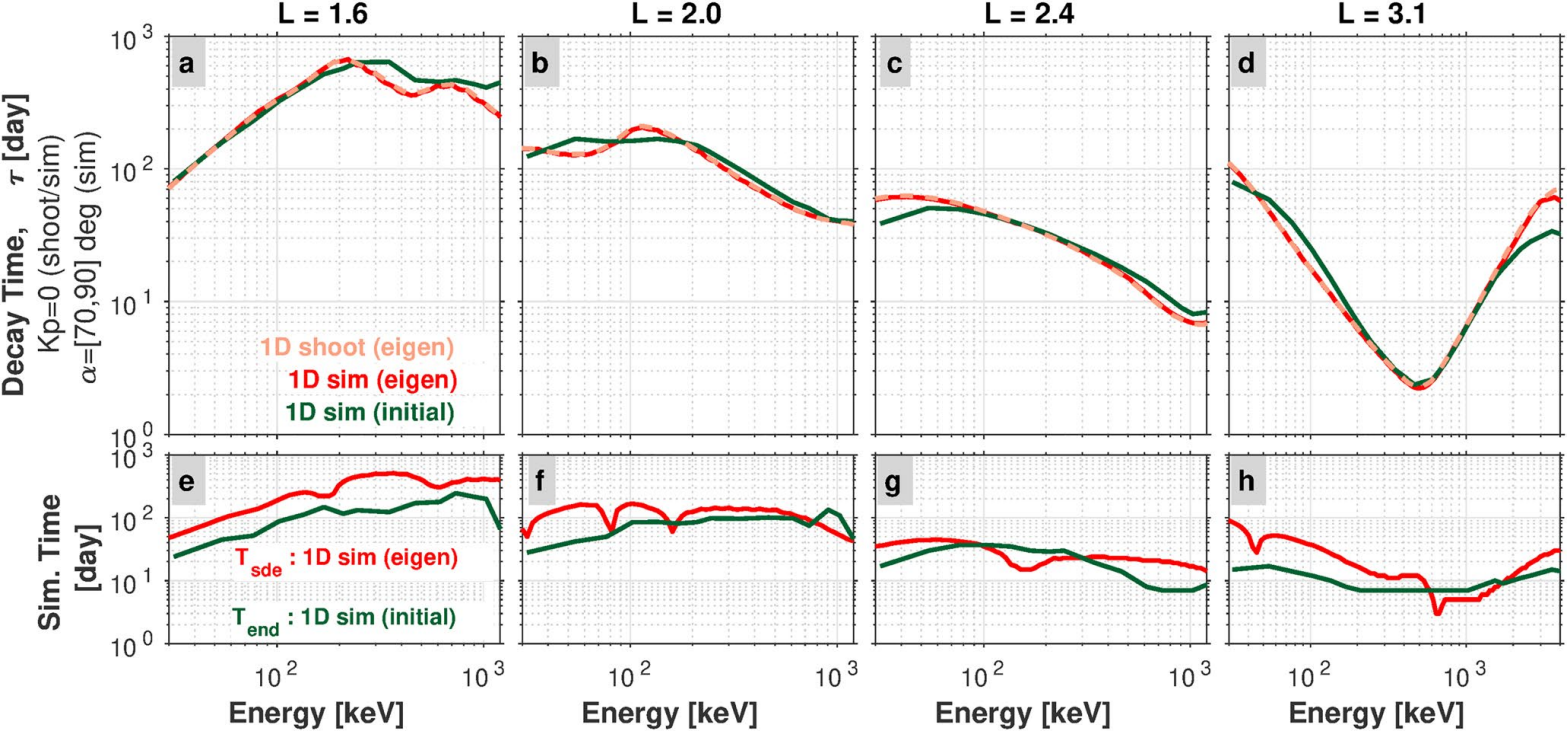
(a)–(d) A comparison of the decay time scales computed from the 1D pitch‐angle diffusion simulations (sim) and those computed directly from the diffusion coefficient via the shooting method (shoot). The curves labeled “eigen” represent the timescales for the slowest decaying eigenmode, while the curves labeled “initial” represent the timescales obtained during the initial part of the decay/simulation. (e)–(h) A comparison of the time step in the simulation at which each state has been reached, either the eigenstate, *T*_*sde*_, or the end of the of the initial part of the decay, *T*_*end*_.

We use this 1% level on the mean relative error in *τ*_*n*_ to obtain the decay timescale (*τ*) and *T*_*sde*_ from the 1D simulations at all energies and at the four *L* values under investigation. These calculations are shown in Figure 7 with the darker red curves. In panel (a), the decay timescale obtained from the simulation using this method is shown in dark red and labeled “1D sim (eigen)” to indicate that it is the eigenvalue of the pitch‐angle diffusion operator. For comparison, we also show the values obtained from the shooting method on Equation 2 (i.e., the values plotted in Figure 2). These are labeled as “1D shoot (eigen)” and are shown with a dashed line in lighter red. The near perfect agreement between the result obtained from the simulation and the result obtained from the shooting method validates our technique of identifying the decay timescales by using the mean relative error on *τ*_*n*_. Panel (e) shows *T*_*sde*_ in red, where we see that the time to reach the equilibrium eigenstate generally increases with increasing energy at this *L*. The subsequent columns in Figure 7 (panels (b) and (f), panels (c) and (g), and panels (d) and (h)) show the same calculations at the other three *L* values under consideration.

#### 3.3.2 Calculating the Decay Timescales From the 2D Simulations

We now return to Figure 6 and the question of how to compute decay timescales from the 2D simulations. Panel (d) shows the simulated fluxes from the 2D simulation without Coulomb energy drag, that is, the solution to Equation 5 with the last term on the right‐hand side omitted. The fluxes are again plotted for the first 700 days of the simulation, as in panel (a), and we see similar behavior as in the 1D simulation. During the initial part of the simulation (up to day ∼100), the fluxes at different pitch angles are all decaying on different timescales. As the simulation progresses, the fluxes begin to settle into a single decay timescale of *τ*_*n*_ ≈ 500 days, reminiscent of the eigenmode in panel (b). However, when viewed on a longer timescale (panels (g) and (h)), we see that, while all pitch‐angles have collapsed into a single decay timescale, this value is time‐dependent. Thus, one cannot assign a single timescale or lifetime to the decaying fluxes in the 2D simulations, as alluded to above. Also, note that in this 2D simulation at this *L* and energy, the flux decays proceed more slowly than in the 1D case subject only to pitch angle diffusion. The additional processes of momentum and cross‐diffusion act to inhibit the decay.

At this point, we turn to the observations as a guide, since we are ultimately trying to use theory to understand what the measurements show. Deep in the inner zone near *L* ≈ 1.5, the decay timescales observed by the Van Allen Probes are long and the fluxes decay over long time intervals (∼100 days), since the decay dynamics are only interrupted by very strong events like the March 2015 and June 2015 geomagnetic storms. However, even with these caveats, it is rare for the fluxes measured in the inner zone to decay in isolation for ∼450 days, as the value of *T*_*sde*_ shown in Figure 7 suggests. It is really the timescale during the “initial” portion of the decay that we are interested in, since this is what is measured. Moreover, this initial portion of the decay is dominated by pitch angle diffusion, since this is the fastest process in the 2D simulation. Thus, we use this guidance from the observations and our theoretical expectations to extract the decay timescales during the initial portions of the simulation, as follows.

First, at each *L* and energy bin, we average the simulated flux over the equatorial pitch angle range from 70° to 90°. We do so because the observed decay timescales were computed using fluxes averaged over roughly the same pitch angle range (Claudepierre et al., 2020b). (We note that the decay timescales obtained from the simulations are not particularly sensitive to this choice of pitch‐angle range; not shown here). Next, we find the first time in the simulation in which this averaged flux decreased for all subsequent days. This value, denoted *T*_0_, is typically within the first ∼10 days of the simulation and marks the beginning of the time interval that we use to calculate the decay timescale. If *T*_0_ is found to be less‐than‐or‐equal to day 3, we impose *T*_0_ = 3 has a hard lower limit, so as to avoid the very initial part of the simulation. The end of this time interval, which we denote as *T*_*end*_, is defined as *T*_0_ plus the observed decay timescale (rounded up to the next integer day). The reason for using the observed decay timescale to specify the upper limit of the time interval is to ensure that we are capturing the decay during the portion of the simulation that is most representative of the time interval over which the decay is observed. In the inner zone, the observed decay timescales are less than ∼200 days, so that the time interval that we use to calculate the decay timescale from the simulation, [*T*_0_, *T*_*end*_], is some subinterval of the first ∼200 days of the simulation. If a value of *T*_*end*_ is found such that the length of [*T*_0_, *T*_*end*_] is less than 5 days, we increase *T*_*end*_ so that the interval length is 5 days. Finally, we fit an exponential to the simulated flux over the time interval [*T*_0_, *T*_*end*_] and retain the e‐folding time as the decay timescale. If the *r* ^2^ of the fit is less than 0.95, we discard the decay timescale and deem it to be undefined. This situation is only encountered in a few bins of *L*‐energy space. It usually arises when the flux is roughly constant and only slightly decaying during the initial part of the time interval, after which time the decay rate increases so that there are effectively two decay timescales within the time interval (and the fit is thus poor).

The technique just described will be used in what follows to calculate the decay timescales from the 2D simulations. Before we do so, we use the 1D pitch angle diffusion simulations to evaluate how the timescales obtained with this technique compare to those of the equilibrium eigenstate. The top row in Figure 7 (panels (a)–(d)) shows the decay timescales obtained from the initial portion of the simulation [*T*_0_, *T*_*end*_], with the label “1D sim (initial)” (green curves). We see that, although the fluxes have not settled into the slowest decaying eigenmode, the timescales obtained from the initial portion of the simulation are quite similar to the eigenstate timescales of pure pitch angle diffusion (the red curves labeled “eigen”). The bottom row in Figure 7 (panels (e)–(h)) compares the time in the simulation in which the eigenstate is reached (*T*_*sde*_) with the end of the time interval over which the “initial” decay timescale is computed (*T*_*end*_). We see that *T*_*end*_ is typically less than *T*_*sde*_, confirming that the calculated decay timescales are obtained from a time interval before the eigenstate is reached. This “initial time interval” method, demonstrated here on the 1D simulations, is used in what follows to compute the decay timescales from the 2D simulations.

#### 3.3.3 2D Simulation Results With and Without Coulomb Energy Drag

**Figure 8:**
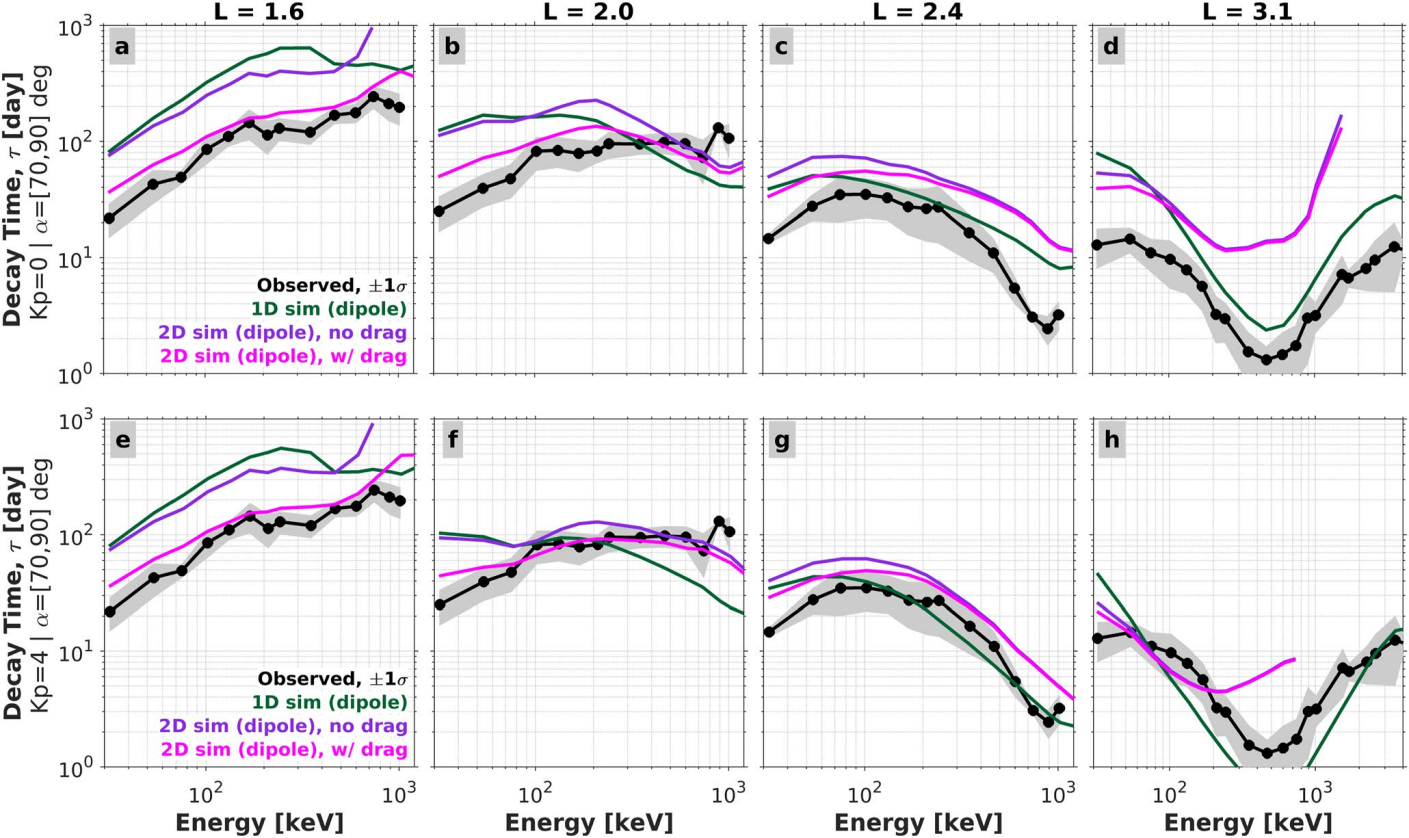
Comparison of the electron decay timescales obtained from 2D simulations with and without Coulomb energy drag (magenta and purple, respectively). Each panel shows the timescales as a function of energy at a fixed *L* value. The top row shows timescales from simulations with *Kp* = 0 diffusion coefficients ((a)–(d)), while the bottom row shows timescales from simulations with *Kp* = 4 coefficients ((e)–(h)). The timescales from the 1D pitch‐angle diffusion simulations are also shown, for comparison (green curves). The mean observed timescales are shown with dotted black curves and the gray‐shaded regions indicate the 1*σ* error on the means.

Figure 8 shows the electron decay timescales from the 2D simulations with and without Coulomb energy drag (magenta and purple curves, respectively). For comparison, the decay timescales from the 1D pitch angle diffusion simulations are also shown (green curves), which are the same green curves shown in Figure 7. Note that, as demonstrated in Figures 7a–7d, these decay timescales obtained during the initial portion of the simulations are a good proxy for the pitch‐angle diffusion eigenmode timescales. This allows us to link back and compare with the results shown in Sections 3.1 and 3.2 (i.e., the eigenmode timescales shown in Figure 2). The theoretical timescales shown in Figure 8 are calculated using the “initial time interval” method described above, with the diffusion coefficients from lifetime model 3 (LM3). The top row shows the timescales obtained using the *Kp* = 0 diffusion coefficients, while the bottom row shows those obtained from the *Kp* = 4 coefficients. The observed decay timescales are shown in black in each panel, with gray shading to indicate the 1*σ* error bars on the means. We note that using the diffusion coefficients from LM4 does not significantly change the lifetimes obtained from the 2D simulation with Coulomb energy drag (not shown here).

At *L* = 1.6 in the top row (panel (a)), we see that the best agreement with the observed decay timescales is achieved in the 2D simulation where Coulomb energy drag is included. The timescales predicted from 1D pitch‐angle diffusion (green) and from 2D momentum/pitch‐angle diffusion (purple) are both much longer than the observed timescales. Note that this is the *L* region identified above where we found the most significant disagreement between the observed lifetimes and those from our 1D pitch angle diffusion lifetime models. The results with energy drag included match well with the observed lifetimes, both in terms of the absolute timescale and its energy dependence. At *L* = 2.0 (panel (b)), a similar result is found, where the incorporation of energy drag modifies the energy dependence such that it is in better agreement with the observed decay timescales. Specifically, by comparing the purple and magenta curves, we see that the influence of the energy drag is more pronounced at lower energies relative to higher energies. This brings the theoretical calculations with Coulomb drag into better agreement with the observed timescales at lower energy. This is consistent with our theoretical expectations, since lower energy electrons will participate in more interactions with free and bound electrons due to their lower velocities, and thus be subject to greater ionization energy loss in the inner region. Collectively, the results shown in panels (a) and (b) suggest that Coulomb energy drag is an important loss process in the *L* < 2 region and should not be neglected in theoretical and numerical treatments of inner zone electrons.

At higher *L* (*L* = 2.4, panel (c)), we see that the influence that Coulomb energy drag has on the decay timescales is less important than at *L* ≤ 2, and only appreciable at energies less than ∼100 keV. At *L* = 3.1 (panel (d)), Coulomb energy drag is unimportant across nearly the entire energy range shown. We note that the timescales from the 2D simulations (purple and magenta) above 1.5 MeV fail the *r* ^2^ goodness‐of‐fit test described, which is why the curves abruptly end there. Also, in panel (d), the scale on the energy axis is extended to 4 MeV, beyond the ∼1 MeV value used for the upper limits in panels (a)–(c). This is because, at this *L*, there are valid observed decay timescales at energies in excess of 1 MeV with which we can compare.

While Coulomb energy drag clearly becomes less important at higher *L*, as expected, there are some unexpected features in the 2D simulations. For example, at *L* = 2.4 (panel (c)), we see that the lifetimes from 1D pitch angle diffusion are lower than those found in the 2D simulations across all energies shown. This suggests that there is enhanced momentum diffusion in the 2D simulations that opposes the losses from pitch angle diffusion, which results in longer lifetimes. A similar result is noted at *L* = 3.1 (panel (d)) across most of the energy range displayed. Also, at this *L*, the decay timescales in the 2D simulations diverge significantly from both the observed values and those from pure pitch angle diffusion. One potential explanation for this behavior could be that enhanced hiss wave activity, such as that which occurs during more geomagnetically active times, is needed to counterbalance the momentum diffusion that suppresses the losses from pitch angle diffusion. For example, at *L* = 3.1 where hiss wave amplitudes reach their maximum values, the statistical hiss wave amplitudes used to calculate our diffusion coefficients are twice as large during active times (*Kp* = 4) versus quiet times (*Kp* = 0; Claudepierre et al., 2020a). Moreover, at this *L*, the observed decay timescales are in the ∼1–10 days range, and we find that these rapid decays generally occur during more active times (not shown here). Thus, one might argue that the *Kp* = 0 diffusion coefficients are not entirely applicable in this *L* region and that the observed decays are subject to a greater influence from enhanced hiss wave activity.

We can test this hypothesis by performing an additional set of simulations using the *Kp* = 4 diffusion coefficients. We note that, of the scattering mechanisms considered in this work, only the hiss wave scattering has a *Kp* dependence (our EMIC wave model also has geomagnetic activity dependence, but at *L* ≤ 3.1, the resonance energy of EMIC waves is generally higher than 4 MeV, and their statistical wave power is weak). The results from the new simulations are shown in the bottom row of Figure 8. At *L* = 1.6 (panel (e)), we see that the decay timescales from the *Kp* = 4 simulations are essentially unchanged relative to the *Kp* = 0 results shown in panel (a). This is expected since scattering from hiss waves is negligible at this *L* due to the small hiss wave amplitudes, and since other scattering processes are more effective here (i.e., Coulomb collisions and VLF transmitter waves). At *L* = 2.4 (panels (c) and (g)), we see that the *Kp* = 0 and *Kp* = 4 theoretical decay timescales are similar at lower energy, whereas at higher energy they are reduced in the *Kp* = 4 case, which brings them into better agreement with the observed. This is due to the enhanced hiss wave scattering, which preferentially affects the higher energy electrons at this *L*.

At *L* = 3.1 (panels (d) and (h)), the decay timescales at lower energy (<200 keV) are in better agreement with the observations in the *Kp* = 4 case. Again, this is due to the enhanced hiss wave scattering, which influences the entire range of energies at this *L*. However, the calculation of the decay timescales in the 2D simulations in the *Kp* = 4 case is complicated by the fact that butterfly distributions begin to form early in the simulation at this *L* (not shown here), due to the presence of momentum diffusion (Albert et al., 2016). Our method to calculate the decay timescales from the simulated fluxes, which uses fluxes averaged over pitch angles from 70° to 90°, is not well suited for this case. In the initial portion of the simulation, the fluxes near 90° pitch angle are decaying, while the fluxes near 70° pitch angle are increasing, forming the butterfly distribution. Because of this, and the averaging over this pitch angle range, the decay timescales computed at energies >200 keV are likely not accurate. Moreover, at energies >700 keV, the exponential fits fail the *r* ^2^ test because of how the butterfly distributions complicate the analysis. Further work will be necessary to investigate this case, which will require comparisons with observed decay timescales at fixed pitch angle. This is beyond the scope of the current study.

The results from the simulations with the *Kp* = 4 diffusion coefficients suggest that enhanced wave scattering during more active times could potentially explain the anomalous features at *L* = 2.4 an *L* = 3.1 in the 2D simulations. However, we emphasize that this argument is only intended to be suggestive, since it is unrealistic for *Kp* to be elevated to 4 for the duration of a decay that proceeds with a characteristic timescale of 10–100 days. In spite of these difficulties in interpreting the 2D simulation results at *L* > 2, we emphasize that the importance of Coulomb energy drag at *L* < 2 has clearly been demonstrated.

## 4 Discussion

The results presented in the previous section are complementary and build upon the work of Albert et al. (2020). We have confirmed their result that Coulomb energy drag is an important scattering process at *L* < 2. An important distinguishing feature between their work and ours is that we make direct comparisons with the observed decay timescales that were obtained by Claudepierre et al. (2020b). In addition, we explicitly calculate decay timescales from the 2D simulations with and without Coulomb drag for comparisons with the observations. This allows for a more comprehensive evaluation of the influence that Coulomb energy drag has on inner belt electron loss timescales, with the observed timescales serving as the ground truth.

However, there are a number of important caveats in our approach to modeling Coulomb drag. For example, we did not simulate a specific event, and simple functional forms were chosen for the initial conditions: ∼sin^2^ *α* for the pitch angle distribution and an exponential for the energy dependence. While these are reasonable choices guided by observations, the Coulomb energy drag term in Equation 5 is sensitive to the parameters used to specify these functional forms. The initial condition on the angular distributions strongly affects the dynamics during the first several days of the simulation and how quickly the distribution approaches and evolves into the lowest eigenmode. Strong injections into the inner zone may be more isotropic than the sin^2^ *α* distribution used here and different dynamics will result. Similarly, the gradients in energy in the distribution function (∼*∂f*/*∂E*) control the overall strength of the Coulomb drag term and the efficiency of momentum diffusion in Equation 5. Future work will be necessary to explore this parameter space on the initial conditions and simulate specific events to fully quantify the role of Coulomb energy drag on inner zone electron dynamics. It is also important to acknowledge the differences in the methodology used here versus that used by Albert et al. (2020). The most notable differences are (a) that we use statistically averaged empirical models for the LGW and VLF waves, while they used a physics‐based calculation, and (b) that we use a plasmasphere density model derived from observations, while they used one based on the theoretical consideration of diffusive equilibrium.

When comparing with the observed timescales, Figure 5 suggests that the unducted propagation mode may be a poor assumption for the LGW waves (for either density model). Albert et al. (2020)'s lifetimes obtained for ducted LGW propagation agree better with the observed lifetimes when their “dens‐high” plasmaspheric model is used. At *L* > 2, this density model is in agreement with the Ozhogin et al. (2012) model that we used, and we find good agreement between the Albert et al. (2020) lifetimes, those obtained in our lifetime model 3 (LM3), and the observed lifetimes. This indicates that we obtained similar results for VLF and LGW wave scattering despite the two different approaches (empirical vs. physics‐based), assuming ducted propagation for the LGW waves in the Albert et al. (2020) results.

At *L* < 2, where we find the largest disagreement between our theoretical calculations (LM3) and the observed decay timescales, we demonstrated that the Albert et al. (2020) lifetimes agree better with the observations. However, we showed that their “dens‐high” plasmaspheric model is inconsistent with both the Ozhogin et al. (2012) density model and the model of Hartley et al. (2018) at *L* < 2. We thus argue that the agreement in lifetimes was solely due to the choice of plasmaspheric density model, which may be artificially large in this region. While the plasmasphere does not typically erode below *L* = 2, some variability in the electron density may be expected based on day/night asymmetries related to the ionosphere. Further work will be needed to fully characterize the electron densities at *L* < 2 and their very important role in controlling electron scattering loss.

It is also important to acknowledge that the equilibrium pitch‐angle diffusion eigenmode state may never be reached in observed electron flux decays in the inner regions (*L* < 4). At lower *L* (*L* < 2), where the decay timescales are long (>100 days) and the decays often proceed uninterrupted, the results presented here suggest that the eigenmode timescale is approached in the observations. However, in the slot region, the decays proceed rapidly, with characteristic timescales on the order of a few days. This may not be of sufficient duration to reach the lowest order eigenmode of the pitch‐angle diffusion operator. We attempted to account for this when calculating the decay timescales from our simulations by only looking at the decay during the initial portion of the simulation. This again highlights the need to carry out event‐specific simulations and compare the observed decay timescales with those simulated to fully assess whether true equilibrium eigenstates are ever realized in inner belt decays.

## 5 Summary

We investigate the factors that contribute to electron precipitation loss in the Earth's radiation belts using the most up‐to‐date wave models and simulation techniques. In our previous work (Claudepierre et al., 2020a, 2020b), we examined electron decay timescales, or lifetimes, in the radiation belt region (*L* = 1.3 to 6). We demonstrated good qualitative agreement between the decay timescales observed by the Van Allen Probes and theoretical calculations based on quasilinear pitch‐angle diffusion. We considered several waves and scattering mechanisms in our diffusion calculations: Scattering from hiss, EMIC, and VLF transmitter waves, and scattering from Coulomb collisions with neutral and charged particles in the atmosphere and ionosphere. While good qualitative agreement was found, quantitative agreement was lacking, particularly in the inner region (*L* < 2.5), where the theoretical decay timescales were found to be roughly an order of magnitude larger than the observed.

In the current study, we have incorporated LGW waves, revised our treatment of VLF transmitter wave scattering, considered the role of the drift loss cone, and evaluated the impact of Coulomb energy drag. The primary findings of this work are summarized as follows:

1. Coulomb energy drag (ionization energy loss) is an important electron loss process in the *L* ≤ 2 region and should not be neglected in theoretical and numerical treatments of inner zone electrons. Including energy drag in our decay timescale calculations significantly improves the quantitative agreement with the observed timescales at *L* = 1.6 and *L* = 2.0.
2. Electron decay timescales in the *L* < 4 region are very sensitive to the choice of plasmaspheric density model (e.g., Albert et al., 2020; Hartley et al., 2018; Ozhogin et al., 2012). For example, theoretical decay timescales at *L* < 1.5 can be brought into quantitative agreement with the observed timescales, without invoking an additional process like Coulomb energy drag, by using a model with electron densities that are a factor of 5–10 larger than the Ozhogin et al. (2012) model at *L* < 1.5.
3. Explicitly incorporating LGW waves into our theoretical lifetime calculations significantly improves the quantitative agreement with the observed electron lifetimes at *L* ≈ [1.8, 3.2], relative to what was presented in Claudepierre et al. (2020a).
4. When the drift loss cone is taken into consideration, lifetimes are reduced by ∼20% at *L* = [1.7, 4.0], by a factor of ∼2–5 at *L* = [1.4, 1.6], and by an order of magnitude or more at *L* < 1.3. This was demonstrated with a simple calculation using the IGRF drift loss cone angle in place of the dipole bounce loss cone angle in our theoretical scattering and lifetime calculations.
5. The lifetimes calculated from our statistically averaged empirical models of LGW and VLF transmitter waves are similar to those obtained using the physics‐based approach of Albert et al. (2020) and Starks et al. (2020).
6. The approximate formula derived by Albert and Shprits (2009) to calculate lifetimes from pitch angle diffusion coefficients produces values ∼2× larger than the exact calculation.
7. The inclusion of LF transmitter wave power in our VLF wave scattering calculations had a minimal impact on the theoretical lifetimes.

The work presented here furthers our understanding of the processes that are relevant for electron loss in the Earth's inner radiation belt region (*L* < 4). These findings will be relevant for future numerical modeling efforts and observations obtained in this important region of geospace.

## Erratum

In the originally published version of this article, two URLs were provided in the Data Availability Statement. The second URL was temporary and has been removed. This version may be considered the authoritative version of record.

## Data Availability

The data displayed in the figures in this manuscript are available at https://doi.org/10.5068/D1FT3H.
